# Novel Pancreatic Cancer Cell Lines Derived from Genetically Engineered Mouse Models of Spontaneous Pancreatic Adenocarcinoma: Applications in Diagnosis and Therapy

**DOI:** 10.1371/journal.pone.0080580

**Published:** 2013-11-20

**Authors:** María P. Torres, Satyanarayana Rachagani, Joshua J. Souchek, Kavita Mallya, Sonny L. Johansson, Surinder K. Batra

**Affiliations:** 1 Department of Biochemistry and Molecular Biology, University of Nebraska Medical Center, Omaha, Nebraska, United States of America; 2 Buffett Cancer Center, Eppley Institute for Research in Cancer and Allied Diseases, University of Nebraska Medical Center, Omaha, Nebraska, United States of America; 3 Department of Pathology and Microbiology, University of Nebraska Medical Center, Omaha, Nebraska, United States of America; Wayne State University School of Medicine, United States of America

## Abstract

Pancreatic cancer (PC) remains one of the most lethal human malignancies with poor prognosis. Despite all advances in preclinical research, there have not been significant translation of novel therapies into the clinics. The development of genetically engineered mouse (GEM) models that produce spontaneous pancreatic adenocarcinoma (PDAC) have increased our understanding of the pathogenesis of the disease. Although these PDAC mouse models are ideal for studying potential therapies and specific genetic mutations, there is a need for developing syngeneic cell lines from these models. In this study, we describe the successful establishment and characterization of three cell lines derived from two (PDAC) mouse models. The cell line UN-KC-6141 was derived from a pancreatic tumor of a Kras^G12D^;Pdx1-Cre (KC) mouse at 50 weeks of age, whereas UN-KPC-960 and UN-KPC-961 cell lines were derived from pancreatic tumors of Kras^G12D^;Trp53^R172H^;Pdx1-Cre (KPC) mice at 17 weeks of age. The cancer mutations of these parent mice carried over to the daughter cell lines (i.e. *Kras^G12D^* mutation was observed in all three cell lines while *Trp53* mutation was observed only in KPC cell lines). The cell lines showed typical cobblestone epithelial morphology in culture, and unlike the previously established mouse PDAC cell line Panc02, expressed the ductal marker CK19. Furthermore, these cell lines expressed the epithelial-mesenchymal markers E-cadherin and N-cadherin, and also, Muc1 and Muc4 mucins. In addition, these cell lines were resistant to the chemotherapeutic drug Gemcitabine. Their implantation *in vivo* produced subcutaneous as well as tumors in the pancreas (orthotopic). The genetic mutations in these cell lines mimic the genetic compendium of human PDAC, which make them valuable models with a high potential of translational relevance for examining diagnostic markers and therapeutic drugs.

## Introduction

Despite many advances in the understanding of molecular mechanisms involved in pancreatic cancer (PC) pathogenesis over the last four decades, the disease remains one of the top malignancies with worst prognosis [1]. These grim statistics are a constant reminder of the urgent need for elucidating yet undiscovered mechanisms of PC pathology that will contribute to improved diagnosis and treatment regimens. For this purpose, developing preclinical models is of vital importance, because they are critical for evaluating novel therapeutic strategies [2]. Xenograft tumors in athymic nude mice are useful preclinical models, but they cannot provide the role of immune mechanisms that may add to or interfere with the action of the therapeutic candidates. More recently, genetically engineered mice (GEM) models that produce spontaneous pancreatic adenocarcinomas (PDAC) have greatly advanced our understanding of PC pathogenesis and also, allowed the examination of novel therapeutic approaches [3]–[6]. In addition, syngeneic cell lines can be isolated from pancreatic tumors produced by GEM models and used for *in vitro* and *in vivo* screening assays. The analysis of functions and characteristics of specific genetic mutations and PC biomarkers present in these cell lines can shed light on the design of promising diagnostic and therapeutic strategies.

Mutations in *KRAS*, *CDKN2A*, *TP53*, and *SMAD4/DPC4* genes are commonly observed in PDAC tumors from PC patients [7]. In consideration of these results, several mouse models that produce spontaneous PDAC, have been engineered in the last decade [3], [4], [6]. The present study focuses on mice carrying *Kras* and *Trp53* mutations. The role of oncogenic *Ras* in PC was examined by directing endogenous expression of *Kras^G12D^* in the progenitor cells of the pancreas in Kras^G12D^;Pdx1-Cre (KC) mice [3], whereas the role of the endogenous expression of *Trp53^R172H^* and *Kras^G12D^* was examined in the pancreas of Kras^G12D^;Trp53^R172H^;Pdx1-Cre (KPC) mice [4]. The results indicate that the spontaneous pancreatic tumors produced by these mouse models recapitulate the clinical, histopathological and genomic features of human PDAC.

Mouse PDAC cell lines with greater clinical relevance to PC are highly needed. The currently available Panc02 cell line has been used over the past three decades [8]. It was derived from PDAC tumors induced by implanting 3-methyl-cholanthrene (3-MCA)-saturated threads of cotton in the pancreas of C57BL/6 mice. Despite its widespread use in evaluating various therapeutic strategies, Panc02 cells lack strong clinical significance for PC due to absence of mutational spectrum when compared to human disease. Consequently, success in translating therapies indicated by this model has been limited. In this manuscript, we describe the generation and characterization of three new PDAC cell lines derived from spontaneous mouse models of PC. One cell line was derived from a KC mouse at 50 weeks age, and two others were derived from KPC mice at 17 weeks of age. The successful establishment and *in vitro* and *in vivo* characterization of these cell lines are comprehensively described, including markers currently known for pancreatic tumors.

## Materials and Methods

### Establishment of Cell Lines

The complete medium consisted of DMEM containing heat inactivated FBS, L-Glutamine (200 mM), 100x non-essential amino acids (100 mM), sodium bicarbonate, HEPES buffer, Gentamicin (50 mg/ml), and Penicillin/Streptomycin (100 µg/ml). Immediately after resecting the tumor, 200 mg of pancreatic tumor tissue was transferred into a petri dish containing sterile PBS and Gentamicin (20 µg/ml).

The collected tissues were washed 3 times with PBS-gentamicin and transferred into a petri dish containing the complete medium. The tumor was finely-minced with a sterile scalpel and transferred into a sterile centrifuge tube with the complete medium containing Collagenase P (Roche, Indianapolis, IN) (10 mg/ml).The mixture was incubated at 37°C for 30 min. The tubes were inverted every three min to ensure proper mixing. Following two washing cycles in complete medium, the pellet obtained after centrifugation was suspended in the complete medium. After letting the tube stand for 2 min, cell suspension without tissue debris was transferred into a new sterile 10 cm Petri dish.

After incubating overnight at 37°C in 5% CO_2_, the medium was replaced with fresh medium. Cells were trypsinized once confluent. To prevent the growth of fibroblasts, differential trypsinization was carried out, where trypsin was added to the cells and after 10 sec, the cells were washed with PBS and fresh medium was added. Additionally, medium was supplemented with cholera toxin (400 ng/ml) for the first 7 passages as it has been previously reported that it has an enhanced mitogenic effect on epithelial cells, but not on fibroblast cells [9]. After 10 passages, the cell lines were transferred to normal complete medium (i.e. DMEM medium supplemented with 10% FBS, 100 µg/ml penicillin, 100 µg/ml streptomycin) and maintained at 37°C and 5% CO_2_ in a humidified incubator. Three cell lines were established successfully. The cell line derived from a Kras^G12D^;Pdx1-Cre (KC) mouse was named UN-KC-6141 and the two cell lines derived from Kras^G12D^;Trp53^R172H^;Pdx1-Cre (KPC) mice were named UN-KPC-960 and UN-KPC-961 (UN designates University of Nebraska Medical Center). *In vitro* characterization and tumorigenic studies were done after 35 passages in cell culture. The murine PDAC cell line Panc02 was included in the *in vitro* functional assays.

### Sequencing of Cell Lines for *Kras* and *p53* Mutations

The sub-confluent cultures of UN-KC-6141, UN-KPC-960, and UN-KPC-961 were lysed with cell lysis solution containing beta-mercaptoethanol and total RNA was isolated with RNeasy Minikit (Qiagen, Germantown, MD). cDNA was synthesized from total RNA using oligo(dT)18 primer and SuperScript II reverse transcriptase (Life technologies™, Carlsbad, CA). PCR amplification of murine *Kras* codon 12 (exon 1) and *p53* codons 172 (Exon 5) was carried out by using set of primers to each gene (*Kras*-seqF:5'-ACTTGTGGTGGTTGGAGCTG-3', *Kras*-seqR:5'-TGACCTGCTGTGTCGAGAAT-3', *p53*-seqF: 5'-CACGTACTCTCCTCCCCTCA-3' and *p53*-seqR: 5'-ATTTCCTTCCACCCGGATAA-3'), which results in a 168 bp (*Kras*) and 229 bp (*p53*) PCR products. The PCR products were purified with PCR product purification kit (Qiagen, Germantown, MD) and the purified PCR products were sequenced using *Kras*-seqR:5'-TGACCTGCTGTGTCGAGAAT-3' for *Kras* and *p53*-seqF: 5'-CACGTACTCTCCTCCCCTCA-3' for *p53* gene.

### Growth Kinetics

For growth kinetics studies, each cell line was seeded in quadruplicate in a 96-well plate (1,000 cells/well) in complete medium. After overnight incubation, the medium was replaced with medium supplemented with 1% FBS and Penicillin/Streptomycin (100 µg/ml). Every day, 10 µl of the Cell Proliferation Reagent WST-1 (Roche, Indianapolis, IN) was added to each well and after 3 h of incubation, absorbance values were measured at 450 nm. Absorbance values were subtracted from values recorded at the reference wavelength (600 nm), as indicated by the manufacturer. The procedure was repeated for 7 days. The doubling time (T_D_) for each cell line was calculated during the exponential growth phase using the formula T_D_ = 0.693t/ln(N_t_/N_0_), where t = time difference in h, N_t_ = absorbance value at time t, and N_0_ = absorbance value at initial time [10].

### Real Time PCR

RNA was isolated and purified from UN-KC-6141, UN-KPC-960, UN-KPC-961, Panc02, and NIH3T3 (mouse fibroblasts) cells using the RNeasy Minikit (Qiagen, Germantown, MD). cDNA was synthesized from total RNA using oligo(dT)18 primer and SuperScript II reverse transcriptase (Life technologies™, Carlsbad, CA) as described by us in a previous publication [11]. Real-time PCR was carried out in the LightCycler 480 (Roche Diagnostics, Indianapolis, IN). The amplification was done in a two-step process (95°C for 5 min followed by 45 cycles of 95°C for 10 sec, 60°C for 10 sec, and 72°C for 10 sec) using the LightCycler 480 SYBR Green I Master Mix (Roche Diagnostics, Indianapolis, IN) and cDNA from each sample. *GAPDH* was used as the internal control gene to which all genes were normalized. The fold-change in gene expression of *Amylase* and *CK19* mRNA in cancer cell lines were compared relative to mRNA levels extracted from normal mouse pancreas using the 2^−ΔΔCt^ method, whereas the fold-change of *Muc1* and *Muc4* were compared relative to mRNA extracted from the mouse fibroblast cell line NIH3T3. The sequence of the primers used in this study are listed in **Table 1**.

**Table 1:** Primer sequences used for real time PCR analysis.

Mouse Gene	Primer Sequence
*Amylase*	FP – 5′-CAAAATGGTTCTCCCAAGGA-3′RP – 5′-ACATCTTCTCGCCATTCCAC-3′
*Cytokeratin 19* (*CK19*)	FP – 5′-ACCCTCCCGAGATTACAACC-3′RP- 5′-CAAGGCGTGTTCTGTCTCAA-3′
*Muc1*	FP-5′-CCCTACCTACCACACTCACGGACG-3′RP-5′-GTGGTCACCACAGCTGGGTTGGTA-3′
*Muc4*	FP-5′-GAGGGCTACTGTCACAATGGAGGC-3′RP-5′-AGGGTTCCGAAGAGGATCCCGTAG-3′
*Muc5AC*	FP-5′-CCTCTCAGAGGAATGTGACTCTGCGC-3′RP-5′-CCAGGCAGCCACACTTCTCAACCT-3′

### Antibodies

The anti-CK19 hybridoma (TROMA-III) developed by Dr. Rolf Kemler was obtained from the Developmental Studies Hybridoma Bank developed under the auspices of the NICHD and maintained at The University of Iowa (Iowa City, IA). Antibody against murine E-cadherin was obtained from Cell Signaling (Danvers, MA). N-cadherin antibody was a kind gift from Dr. Keith R. Johnson from UNMC (Omaha, NE). β-actin and Amylase antibodies were purchased from Sigma Aldrich (St. Louis, MO). For confocal studies the secondary antibodies conjugated to Alexa Fluor® (568, 488) were obtained from Life Technologies™ (Carlsbad, CA). The secondary antibodies conjugated to horseradish peroxidase used for western blot analysis were obtained from GE Healthcare Life Sciences (Uppsala, Sweden). Anti-mouse Muc1 antibody (mouse monoclonal antibody recognizing the cytoplasmic tail of Muc1) was purchased from Abcam® (Cambridge, MA, USA). The anti-Muc4 (4A-rabbit polyclonal) antibody was designed in this lab and developed by GenScript (Piscataway, NJ, USA) as has been described previously [12].

### Confocal Microscopy

Protein expression was analyzed by confocal microscopy. 2×10^5^ cells suspended in complete medium were seeded on glass cover slips placed in a 12-well plate. The next day, cells were fixed in ice cold methanol. After washing in PBST and blocking with 10% goat serum (Jackson Immunoresearch Labs, West Grove, PA), the cells were incubated with antibodies for Amylase (1:500), CK19 (1:1,000), E-cadherin (1:50), N-cadherin (1:20), and Muc1 (1:100) overnight. They were washed four times with PBST, 5 min per wash. The cells were incubated with the respective secondary antibodies (mouse/rabbit) conjugated to Alexa Fluor (568, 488) (Life Technologies™, Carlsbad, CA) and after repeating the washing steps, the glass coverslips were mounted on glass slides with vectashield mounting medium (Vector Laboratories, Burlingame, CA). The cells were imaged on a laser confocal microscope LSM 510 (Carl Zeiss GmbH, Thornwood, NY) in the respective wavelengths at a magnification of ×63.

### Western Blot Analysis

For western blot analysis, cells were seeded on each well of a 6-well plate in complete medium. At ∼80% confluency, cells were lysed with radioimmunoprecipitation assay buffer (RIPA) containing protease and phosphatase inhibitors. The lysates were subjected to several freeze-thaw cycles to ensure complete lysis. The concentration of lysates was determined with the micro–bicinchoninic acid protein estimation kit (Bio-Rad, Hercules, CA). The protein concentrations were adjusted and solutions were prepared under reducing conditions (i.e. β-mercaptoethanol). 2% SDS-agarose gels were used for analysis of Muc4 expression. 40 µg of protein lysates were loaded and the gel was run for 4 h at 100 V. E-cadherin and N-cadherin expression levels were analyzed by SDS-PAGE. 40 µg of protein lysates were loaded and separation was done at 80 mA for 1 h. Resolved proteins were transferred onto polyvinylidene difluoride membranes, blocked in 5% milk in PBS, and incubated with the primary antibodies overnight. After washing with PBST, the corresponding secondary antibodies were added and after repeating the washing steps, the proteins were detected by luminol (Thermo Scientific, Middletown, VA) after exposure to X-ray films.

### Cytotoxic Assay

The cytotoxic effect of the chemotherapeutic drug Gemcitabine on KC and KPC cell lines was compared to the cytotoxic effect on Panc02. The injectable solution of Gemcitabine, GEMZAR® (Eli Lilly Company, Indianapolis, IN) was kindly provided by the pharmacy at the Lied Transplant Center at UNMC. For the cytotoxic assay, 1×10^4^ cells suspended in complete medium were seeded in each well of a 96-well plate. The next day, the cells were treated with different concentrations of Gemcitabine solution (100 nM-100 µM) in quadruplicate wells. After incubating the cells with Gemcitabine for 48 h, media containing thiazolyl blue tetrazolium bromide reagent (Sigma Aldrich, St Louis, MO) was added to the cells. After 4 h of incubation, the formazan crystals produced by metabolically active cells were dissolved with 100 µl of DMSO. Absorbance values at 540 nm were used to calculate cytotoxicity percentages. The half maximal inhibitory concentration (IC-50) of Gemcitabine was determined in each cell line from interpolating values in the graph (% Cytotoxicity vs. Gemcitabine Concentration).

### Tumorigenicity studies

The tumorigenicity of the cell lines was determined after orthotopic implantation of the mouse PC cell lines (UN-KC-6141/UN-KPC-960/UN-KPC-961) into the head of the pancreas after 35 passages. Based on the mice background from where the cell lines were generated, UN-KC-6141 cells (1×10^6^) were injected in C57BL/6 mice, whereas UN-KPC-960 and UN-KPC-961 cells (1×10^6^) were injected in mixed background (i.e. B6.129) mice (N = 7). Mouse PC cells suspended in 50 µl sterile PBS were injected orthotopically using the same procedure described by us [13], [14]. Subcutaneous tumor growth was evaluated with the UN-KPC-961 cell line only. 5×10^6^ cells were injected (N = 12) in mixed B6.129 background mice on the lateral chest. Tumor growth was monitored by palpation/Vernier caliper measurements in case of subcutaneous tumors. Throughout the experiment, animals were provided with food and water ad libitum and subjected to a 12-h dark/light cycle. Animal studies were performed in accordance with the U.S. Public Health Service "Guidelines for the Care and Use of Laboratory Animals" under an approved protocol by the University of Nebraska Medical Center Institutional Animal Care and Use Committee (IACUC). After euthanization, pancreatic tumors were dissected out, weighed and fixed in 10% formalin (Fisher Scientific, Fair Lawn, NJ) for H&E staining. Gross metastatic lesions were examined in distant organs and processed for histological analysis.

### Hematoxylin and eosin staining (H&E)

After fixing tissues in 10% formalin for at least 48 h, the tissues were embedded in paraffin and serial tissue sections (4 μm thick) were cut. The sections were deparaffinized using EZ-DeWaxTM (Bio genex, San Roman CA, USA) and rehydrated progressively. Afterwards, the sections were stained with hematoxylin and eosin (H&E) stains and examined by a certified pathologist.

### Statistics

Significant differences in the experimental values were determined by calculating p-values using the JMP® Statistical Discovery Software (Cary, NC). A Student’s t-test was used to calculate the corresponding *p-value* (*p-values* < 0.05 were considered statistically significant).

## Results

### *In vitro* establishment of KC and KPC cell lines

**Figure 1:**
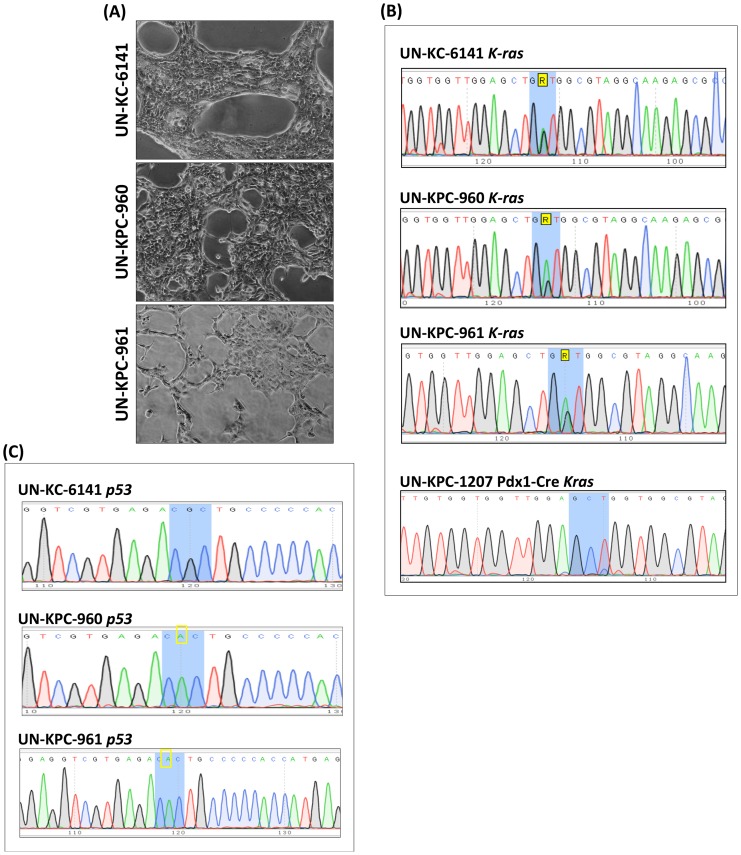
*In vitro* establishment of KC and KPC cell lines. (**A**) Inverted microscope images (4X) of UN-KC-6141, UN-KPC-960, and UN-KPC-961 PDAC cell lines after 35 passages. The three cell lines displayed typical cobblestone epithelial morphology. Genetic sequence analysis of (**B**) *Kras^G12D^* and (**C**) *Trp53^R172H^* mutation in mouse PC cell lines. Blue shaded areas represent the codon where the mutation is located. A yellow rectangle indicate the nucleotide responsible for the mutation. A pancreas from a Pdx1-Cre mouse (mouse tag UN-KPC-1207) was used as a negative control for *Kras* mutation.

Mouse PC cell lines were successfully generated from the spontaneous pancreatic tumors produced by the Kras^G12D^;Pdx1-Cre (KC) and the Kras^G12D^;Trp53 ^R172H^;Pdx1-Cre (KPC) mice at 50 weeks and 17 weeks of age, respectively. One cell line derived from the KC mice was named UN-KC-6141, and two other cell lines derived from the KPC mice were named UN-KPC-960 and UN-KPC-961. All three cell lines grew for over 50 passages in normal media with no sign of senescence. Monolayer cultures of all three cell lines showed typical cobblestone morphology with closed contact island formation (**Fig 1A**). To verify that these cell lines maintained the genetic mutations of their respective parental mice (KC and KPC), the mutational status of the *Kras* (**Fig. 1B**) and *Trp53* (**Fig. 1C**) loci was examined. As expected, all three cell lines carried the *Kras^G12D^* point mutation, whereas UN-KPC-960 and UN-KPC-961 carried the *Trp53^R172H^* activating mutation.

**Figure 2:**
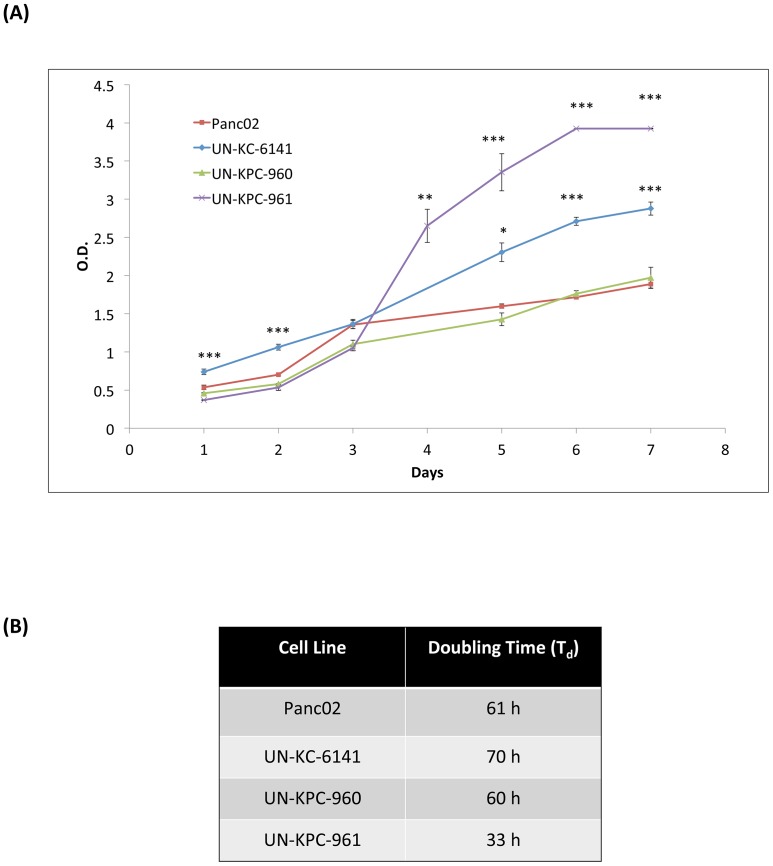
Growth kinetics of mouse PDAC cell lines. (**A**) Cells were seeded in quadruplicate wells of 96-well plates and their growth was followed every day by measuring absorbance after incubation with WST-1 reagent. Data is represented as the mean absorbance (λ_Sample_  =  450 nm, λ_Ref_  =  600 nm) of four replicates ± standard error. Statistics were calculated in comparison to the growth curve for Panc02 (*p < 0.01, **p < 0.001, ***p < 0.0001). (**B**) Doubling time (T_D_) of cell population was calculated using the equation T_D_ = (0.693t)/ln(N_t_/N_0_) where t  =  time difference in h during log phase, N_t_  =  absorbance value at time t, and N_0_  =  absorbance value at initial time.

The growth kinetics of these three mouse PC cell lines were compared with Panc02 cells (**Fig 2A**). UN-KPC-961 cells proliferated the fastest with a doubling time (T_D_) of 33 h (*p* < 0.0001 compared to Panc02). UN-KPC-960 and Panc02 grew at similar rates (T_D_ ≈ 60 h), while UN-KC-6141 showed the slowest growth rate (T_D_ of 70 h) (**Fig 2B**).

### KC and KPC-derived cell lines have ductal-like characteristics

**Figure 3:**
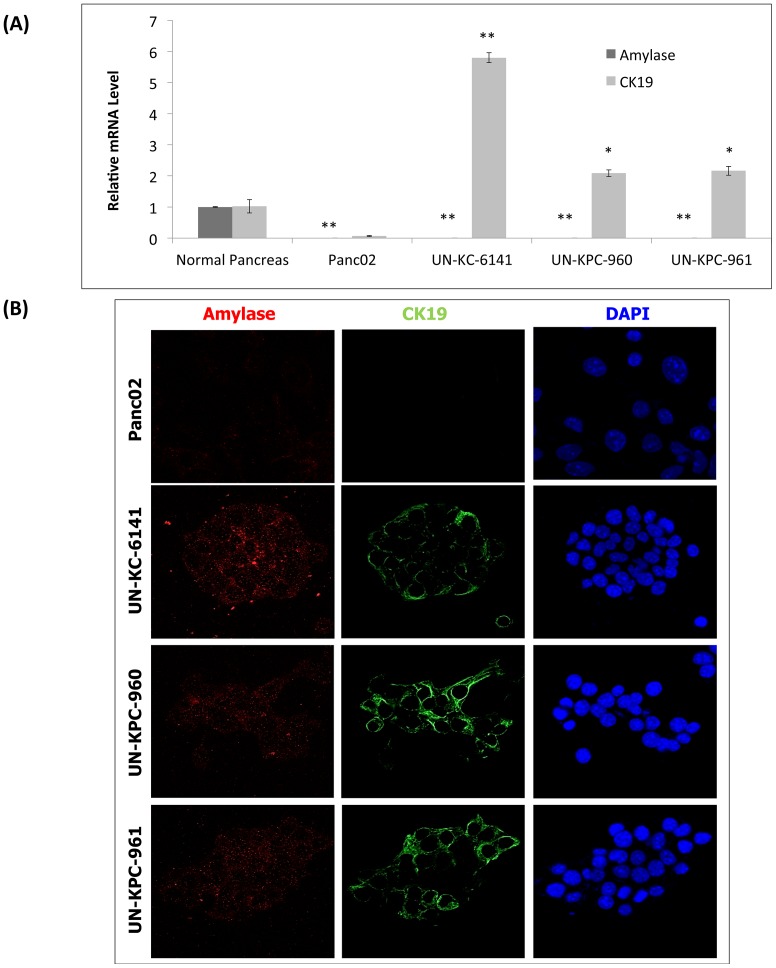
Ductal characteristics of KC and KPC cell lines. (**A**) Real-time PCR analysis of *Amylase* and *CK19* in mouse PDAC cells. These mRNA transcripts were compared relative to the mRNA levels in normal pancreas. The data represents the mean fold increase of three replicates ± standard error. Statistics were calculated in comparison to normal pancreas (*p < 0.005, **p < 0.0001). (**B**) The protein expression of Amylase and CK19 were evaluated on mouse PDAC cell lines by confocal analysis. Amylase was visualized after staining with a secondary antibody conjugated to Alexa Fluor® 568 (Red Fluorescent) and CK19 was visualized after staining with Alexa Fluor® 488 (Green Fluorescent). Cell nuclei were stained with DAPI.

PDACs are thought to arise from the epithelial cells of the pancreatic duct [7]. The exocrine pancreas contains both acinar and ductal cells. Pancreatic acinar cells are characterized by amylase expression and lack of expression of either cytokeratin 19 (CK19) or mucins, and the ductal cells are characterized by expression of CK19 and mucins but no expression of amylase [15], [16]. Indeed, previous studies have reported pancreatic tumors from KC and KPC mice expressed CK19 and frequently, mucin, indicating their ductal heritage [3], [4]. To validate whether our KC and KPC derived-cell lines had these ductal characteristics, we examined amylase and CK19 expression by real time PCR and confocal microscopy. Panc02 cells were included in these analyses for comparison, and normal pancreatic tissue was used for estimating relative gene expression. Real-time PCR experiments showed high expression of the CK19 ductal marker in our three new PDAC cell lines (*p* < 0.005) compared to Panc02 (**Fig. 3A**). To our surprise, Panc02 cells did not show any CK19 expression, even though it has been referenced as a murine ductal PDAC cell line for nearly three decades [8]. None of the four cell lines showed amylase expression, whereas normal pancreas expressed this acinar marker. These results were further confirmed by confocal microscopy analysis (**Fig. 3B**).

### KC and KPC cell lines have epithelial-mesenchymal characteristics

**Figure 4:**
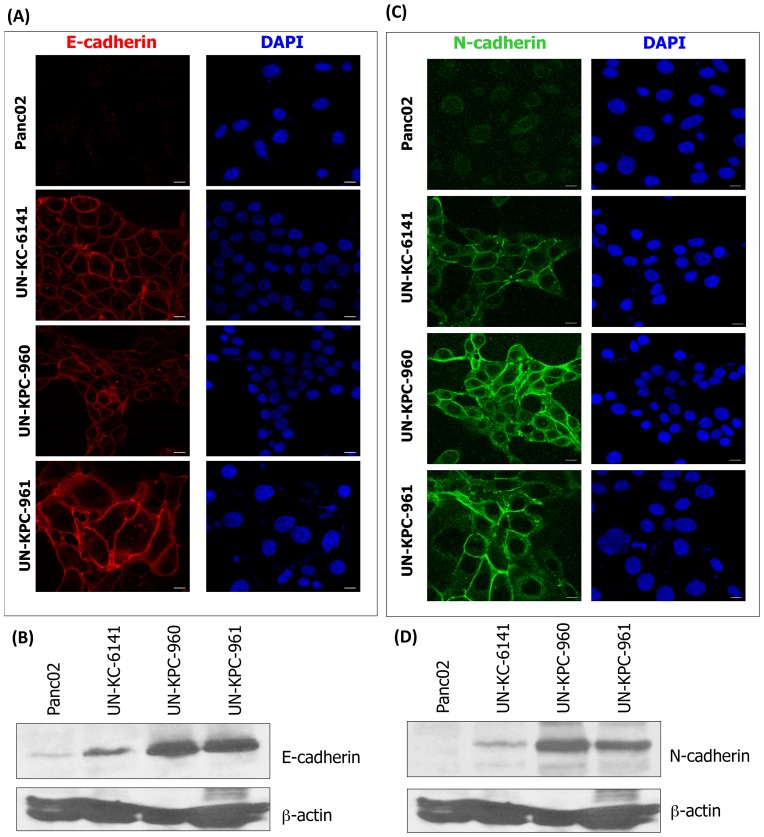
Epithelial-mesenchymal characteristics of KC and KPC cell lines. (**A**) Confocal microscopy images of E-cadherin (Alexa Fluor® 568, Red Fluorescent) expression in mouse cell lines. Cell nuclei were stained with DAPI. (**B**) Western blot analysis of E-cadherin in mouse cell lines. Protein lysates were resolved by 10% SDS-PAGE. β-actin was used as loading control. (**C**) Confocal microsocopy images of N-cadherin (Alexa Fluor® 488, Green Fluorescent) expression in mouse cell lines. Cell nuclei were stained with DAPI. (**D**) Western blot analysis of N-cadherin in mouse cell lines. Protein lysates were resolved by 10% SDS-PAGE. β-actin was used as loading control.

The epithelial-mesenchymal transition induces loss of cell adhesion, which corresponds to E-cadherin downregulation and increased expression of N-cadherin, leading to the initiation of PDAC metastasis [17]. We examined E-cadherin and N-cadherin expression by confocal microscopy and western blot analyses in the three newly-established KC and KPC cell lines. E-cadherin expression was observed in UN-KC-6141, UN-KPC-960, and UN-KPC-961 cells, indicative of their epithelial nature (**Fig. 4A-B**). In contrast, Panc02 cells showed minimal E-cadherin expression. The pattern of N-cadherin expression was similar, being more prominent in the KC and KPC cell lines, compared to the expression in Panc02 cells (**Fig. 4C-D**).

### KC and KPC cell lines express high levels of Muc1 and Muc4

**Figure 5:**
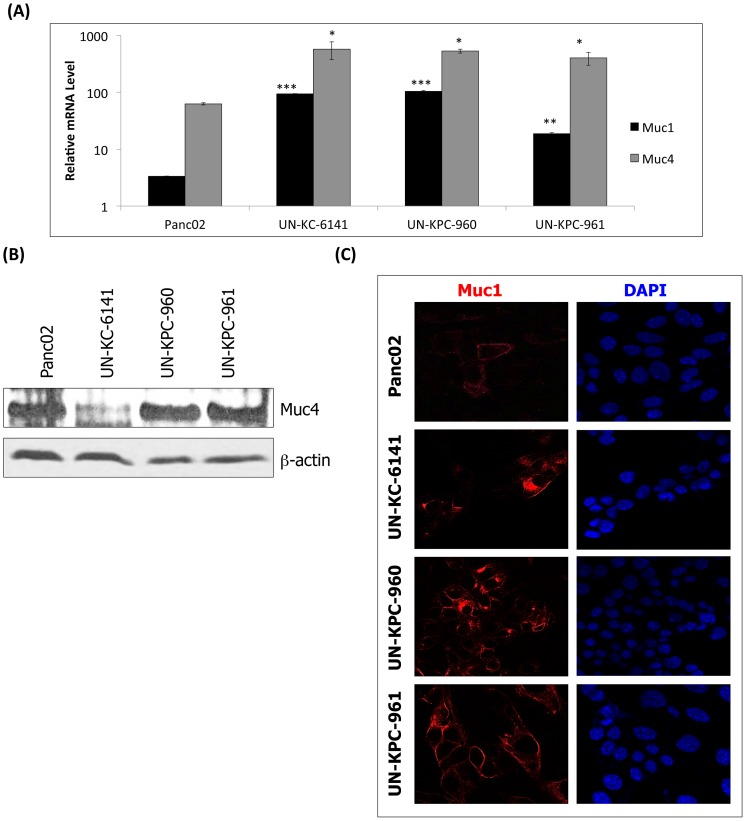
Mucin expression in mouse PDAC cell lines. (**A**) Real time PCR analysis of *Muc1* and *Muc4* in mouse PDAC cell lines. The mRNA transcripts were normalized to mRNA levels in the mouse fibroblast cell line NIH3T3. The data represents the mean fold increase of three replicates ± standard error. Statistical significances were calculated in comparison to NIH3T3 (*p < 0.05, **p < 0.001, ***p < 0.0001). (**B**) Western blot analysis of Muc4 in mouse cell lines. Protein lysates for Muc4 analysis were resolved by 2% SDS agarose gels. β-actin was used as a loading control and it was resolved in 10% SDS-PAGE. (**C**) Confocal microscopy images of Muc1 (Alexa Fluor® 568, Red Fluorescent) expression in mouse cell lines. Cell nuclei were stained with DAPI.

Different types of mucins are expressed in the pancreas during PC progression [12], [18]. Furthermore, PC precursor lesions in genetically engineered mouse PDAC models such as the KC and KPC mice, are known to produce mucins [3], [4]. Recently, we also have demonstrated that in the pancreas of KC mice, expression of Muc1, Muc4, and Muc5AC progressively increased in correlation with PDAC development [12]. We examined whether the expression of these mucins can be corroborated in the KC and KPC-derived cell lines. To this end, real-time PCR, western blot, and confocal microscopy analyses were conducted. The results indicated that relative transcript levels of the transmembrane mucins *Muc1* (*p* < 0.001) and *Muc4* (*p* < 0.05) were significantly higher in UN-KC-6141, UN-KPC-960 and UN-KPC-961 cells (**Fig. 5A**) compared to control cells (NIH3T3 fibroblasts). In Panc02 cells, the relative transcript levels of *Muc1* and *Muc4* were elevated, but they were not statistically significant. Nevertheless, Panc02 cells and the two cell lines derived from KPC mice showed higher levels of Muc4 protein than the KC-derived cell line (**Fig. 5B**). On the other hand, Muc1 protein in Panc02 was lower than in the KC and KPC-derived cells (**Fig. 5C**). To our surprise, Muc5AC was not detectable in any of the cell lines, either at transcript or at protein levels (data not shown), and this may be a consequence of evolutionary changes associated with cell culture.

### KC and KPC cell lines are resistant to Gemcitabine

**Figure 6:**
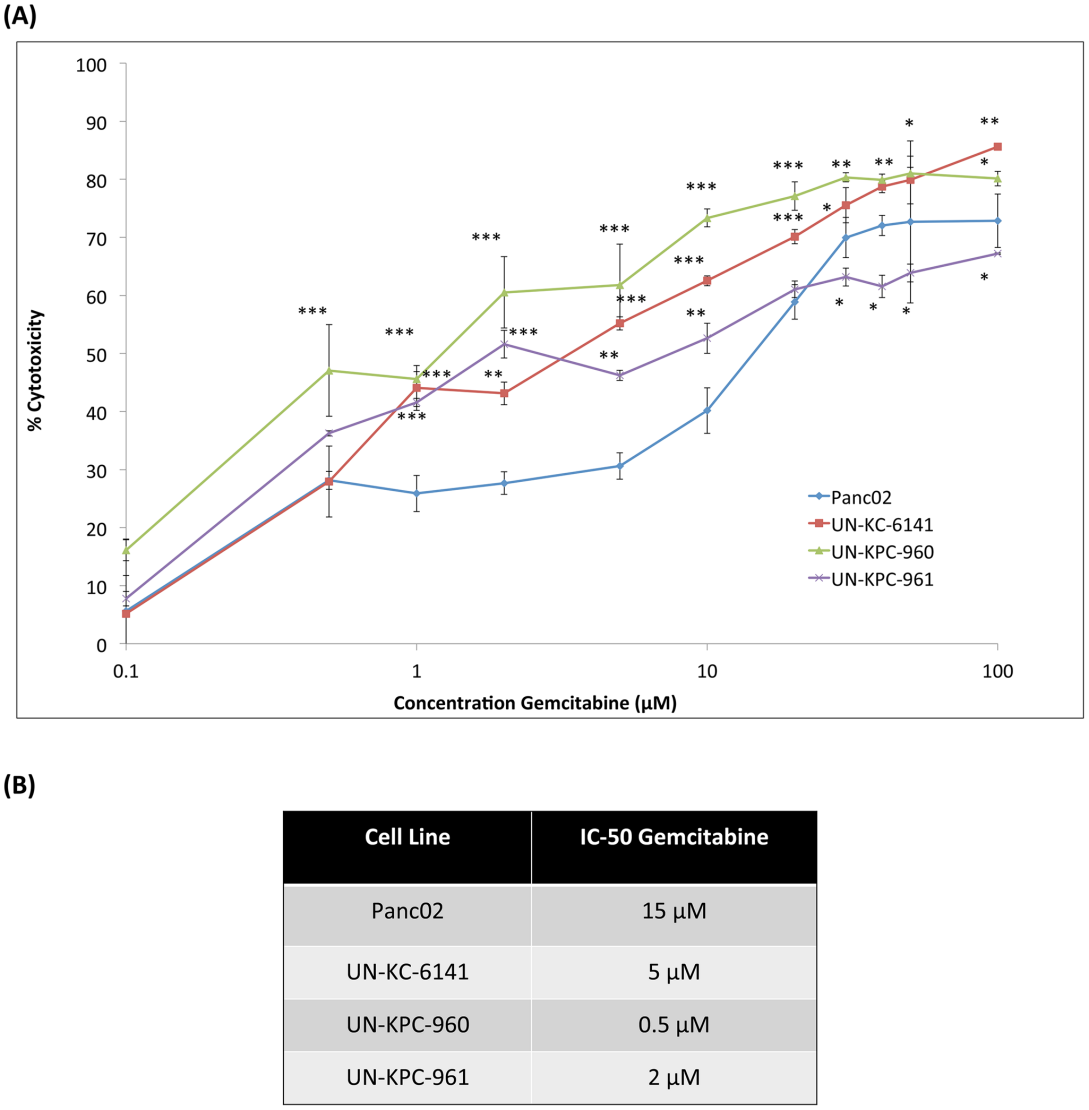
Cytotoxic effects of Gemcitabine in mouse PDAC cell lines. (**A**) Gemcitabine cytotoxicity in mouse PDAC cell lines was determined by the MTT cytotoxic assay. Cells were seeded in quadruplicate wells and incubated with different concentrations of Gemcitabine (100 nM–100 µM) for 48 h. After replacing media with the MTT reagent and dissolving the formazan crystals with DMSO, cytotoxicity was calculated based on the absorbance values (λ  =  540nm) in cells treated with media only. The presented data are average of cytotoxicities in quadruplicate wells ± standard error. Statistical significance was calculated in comparison to Panc02 (*p < 0.01, **p < 0.001, ***p < 0.0001). (**B**) The half maximal inhibitory concentration (IC-50) of Gemcitabine in each cell line was determined after interpolation in the graphs of %Cytotoxicity vs. Concentration.

Despite recent reports that PC patients show greater survival when treated with FOLFIRINOX (a combination of oxaliplatin, irinotecan, fluorouracil and leucovorin) instead of Gemcitabine [19], the latter is still the most used drug in PC chemotherapy [20]. Therefore, using the MTT assay, we examined Gemcitabine cytotoxicity on UN-KC-6141, UN-KPC-960, UN-KPC-961 and Panc02 cells (**Fig. 6A**). Panc02 cells were the most Gemcitabine-resistant with a half maximal inhibitory concentration (IC-50) of 15 µM after 48 h of treatment (**Fig. 6B**). The IC-50 values of the KC and KPC cell lines ranged from 0.5 µM to 5 µM. Although Panc02 cells were more resistant to Gemcitabine, these IC-50 values are in the comparable range of human PC cells [21], [22]. Interestingly, at higher Gemcitabine concentrations (30–100 µM) (**Fig. 6A**), the UN-KPC-961 cells showed greater Gemcitabine resistance than the Panc02 cells.

### Transplanted KC and KPC cell lines induce pancreatic tumors in mice

**Figure 7:**
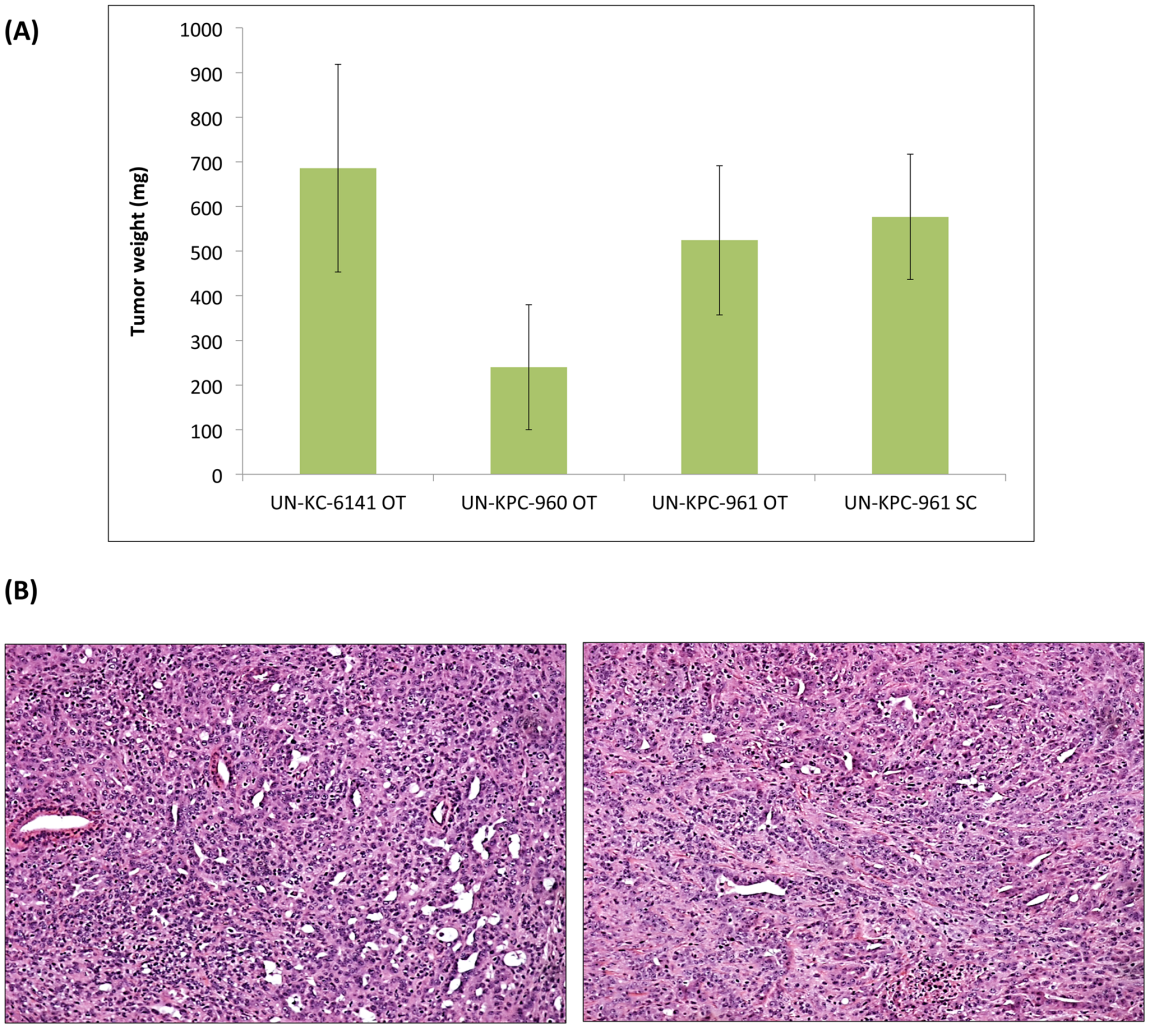
Tumorigenicity of KC and KPC cell lines. (**A**) The tumorigenic properties of UN-KC-6141, UN-KPC-960, and UN-KPC-961 cells were evaluated after orthotopic (OT) (N = 7) implantation of 1×10^6^ cells in the respective mice background. Subcutaneous (SC) injections of UN-KPC-961 cells (5×10^6^ cells) were also performed. OT tumors were grown for different time intervals: one month for UN-KC-6141 and two months for UN-KPC-960 and UN-KPC-961. SC tumors were grown for three weeks. Data is represented as the average weight of pancreatic tumors ± standard error. Differences in tumor size after mice euthanization were not statistically significant. (**B**) Hematoxylin & eosin stained tumor sections (10X) from UN-KPC-961 cells after OT implantation (left) and SC implantation (right). The tumors presented characteristics of poorly differentiated adenocarcinoma.

The establishment of the KC and KPC cell lines in culture provided the opportunity to examine their tumorigenicity in appropriate mouse background from which they were derived. The UN-KC-6141 cells were tested in the C57BL/6 mice, while UN-KPC-960 and UN-KPC-961 cells were examined in mice of mixed B6.129 background. One month after orthotopic implantation of UN-KC-6141 cells in C57BL/6 mice, tumors formed in their pancreas (**Fig. 7A**), and metastatic lesions in liver, spleen, small intestines, mesenteric lymph nodes and peritoneal wall were observed. Tumor incidence for UN-KC-6141 cells was 100%. The two KPC-derived cells (UN-KPC-960 and UN-KPC-961) also formed tumors after orthotopic implantation (**Fig. 7A**), but those appeared at slower rates and the tumors took over two months to grow. The comparatively slower tumor growth kinetics of the KPC cells may have been caused by genetic differences between KC and KPC cells, as well as the different backgrounds of their respective host mice. Interestingly, the UN-KPC-961 cells appeared to be more tumorigenic (tumor incidence 86%) than the UN-KPC-960 cells (tumor incidence 43%). These results are in agreement with their growth kinetics (i.e. UN-KPC-961 showed faster growth than UN-KPC-960). In addition, UN-KPC-961-derived tumors appeared to be more aggressive, showing metastasis to spleen, stomach, liver, and diaphragm, whereas the UN-KPC-960-derived tumors did not show any metastatic lesions. When UN-KPC-961 PC cells were injected subcutaneously into the mixed B6.129 background mice, all mice developed tumors in three weeks, but no metastases were observed. **Figure 7A** shows a comparison of tumors derived from these experiments. Although the average weights of the orthotopic tumors derived by the UN-KPC-960 cells were smaller, tumor weight differences across all groups were not statistically significant. Further, we also performed subcutaneous injection using UN-KC-6141 and UN-KPC-960 PC cells into two C57BL/6 and B6.129 background mice and all the mice developed tumors after 4 weeks (data not included).

For PDAC, histological grading is assigned by the extent of glandular differentiation [23]. If over 95% of a tumor is composed of glands then it is classified as well differentiated, if that extent is 50–95%, a tumor is considered moderately differentiated, and if less than 50%, a tumor is described as poorly differentiated. Histologic analyses classified the KC and KPC-derived tumors as moderately to poorly differentiated tumors, because they did not show duct formation (**Fig. 7B**). In fact, most of these tumors presented characteristics of poorly differentiated (grade III) adenocarcinoma. These results are different from previous studies reporting that most spontaneous pancreatic tumors from KC and KPC mice show moderately well-differentiated to well-differentiated morphology [3], [4].

## Discussion

Genetically engineered mouse models are presently a promising approach for understanding the pathogenesis and progression of PDAC and for evaluating novel therapeutic agents (natural/synthetic) that should translate to high clinical success [24]. Unfortunately, the generation and maintenance of these advanced models require a high cost and time investment. PDAC cell lines from these model mice should have a major role in facilitating screening and prioritization of the variables (e.g. study gene roles or novel therapies), before the ideas are evaluated in the more expensive mouse models.

To our knowledge, this is the first paper reporting the successful establishment and characterization of PDAC cell lines derived from Kras^G12D^;Pdx1-Cre (KC) and Kras^G12D^;Trp53^R172H^;Pdx1-Cre (KPC) mouse models. The three cell lines derived in these studies, named UN-KC-6141, UN-KPC-960, and UN-KPC-961, have been maintained in cell culture for over 50 passages without any sign of senescence. These cell lines show typical epithelial cobblestone morphology, and expressed high levels of epithelial and mesenchymal markers such as CK19, E-cadherin, N-cadherin, Muc1, and Muc4 (**Table 2**). Although Panc02 has been classified as a PDAC cell line on the basis of histological analysis since its establishment three decades ago [8], we noted with interest that it does not express the ductal marker CK19, which raises questions about its phenotype (**Table 2**). Furthermore, unlike the KC and KPC cell lines, Panc02 cells did not express E-cadherin and N-cadherin. As differential levels of these cadherins have a major role in metastasis [17], these cell lines should be useful for evaluating various therapeutic agents or study the fuction of various genes by manipulation.

**Table 2:** Summary of protein expression in murine PDAC cell lines.

Cell Line	Amylase	CK19	E-cadherin	N-cadherin	Muc1	Muc4	Muc5AC
Panc02	–	–	–	–	+	+	–
UN-KC-6141	–	+	+	+	+	+	–
UN-KPC-960	–	+	+	+	+	+	–
UN-KPC-961	–	+	+	+	+	+	–

The most commonly occurring mutations in PDAC include those in the *KRAS*, *CDKN2A*, *TP53*, and *SMAD4/DPC4* genes [7]. Mutations in the *KRAS* gene are present in 90% of PDAC [25], *TP53* mutations occur in ∼75% of PDAC [26], and 55% of pancreatic tumors have *SMAD4/DPC4* deletions or mutations [27]. The KC and KPC-derived cell lines described here did not suffer from evolutionary changes in their cancer genes. Sequencing of these cell lines for point mutations in *KRAS* and *P53* genes revealed the presence of *Kras* point mutations (Gly-Asp) in all the KC and KPC-derived cell lines, and the *Trp53* mutation (R172H) was only present only in the KPC cell lines. Two recent publications have reported the well-established Panc02 cell line has a *Smad4* mutation, but both *Kras* and *Trp53* mutations are absent [28], [29]. Considering that human PDAC show higher frequency of *KRAS* and *TP53* mutations and ductal characteristics, the newly derived murine PDAC cell lines UN-KC-6141, UN-KPC-960, and UN-KPC-961 should be very valuable preclinical studies and have excellent translational significance.

Gemcitabine is the most used chemotherapeutic treatment for PC patients, but tumors may acquire resistance, as evidenced by the poor survival statistics [30]. Some studies have shown that epithelial to mesenchymal transition contributes to drug resistance in PC cells [31], [32]. In one of these studies it was documented that E-cadherin levels in cancer cells have an inverse correlation with drug resistance [32]. This may explain why Panc02 was more resistant to the effects of Gemcitabine (IC-50  =  15 µM) when compared to the spontaneous PDAC cell lines (IC-50s  =  0.5–5 µM). The expression levels of E-cadherin in UN-KC-6141, UN-KPC-960, and UN-KPC-961 cells correlated with decreased drug resistance. It is important to note that even though the KC and KPC-derived PDAC cell lines were not as resistant to Gemcitabine as the Panc02 cells, these cytotoxicity levels are in the range of those found in human PDAC cell lines [21], [22].

In conclusion, the UN-KC-6141, UN-KPC-960, and UN-KPC-961 cells described here represent a valuable tool for yielding a better understanding of PC pathogenesis and to study novel treatments for this lethal disease. The genotypic and phenotypic characteristics of these novel cell lines mimic human PDAC, which make them valuable models with significant translational relevance.
